# Artificial intelligence-assisted feedback in pharmacology education: a pilot evaluation of a custom generative model

**DOI:** 10.1186/s12909-026-09300-w

**Published:** 2026-05-30

**Authors:** Edward Stephenson, Katie Morrigan, Ayesha Irfan, Kate Bascombe, Michael Okorie

**Affiliations:** 1 Department of Medical Education, Brighton & Sussex Medical School, Brighton, UK; 2 Royal Sussex County Hospital, University Hospitals Sussex NHS Foundation Trust, Brighton, UK

**Keywords:** Generative AI, Pharmacology education, Assessment feedback, Large language models

## Abstract

**Background:**

Timely, individualised feedback is central to effective learning in medical education but remains resource intensive, particularly when based on short answer questions (SAQs). Large language models (LLMs) offer potential to support feedback provision, yet prospective evaluations of their accuracy and educational value remain limited. This pilot study evaluated the feasibility, accuracy and student perceptions of AI-generated pharmacology SAQ feedback.

**Methods:**

A prospective pilot study was conducted within a UK Physician Associate MSc programme between March and October 2025. Students were invited to complete voluntary four-question formative pharmacology SAQs. A bespoke custom Generative Pre-trained Transformer (GPT), built using GPT-4 family LLM, guided by a standardised rubric and references, generated structured feedback. All outputs underwent faculty moderation prior to release. Student perceptions were assessed using a 5-point Likert-scale survey. 25% of student responses were double marked and independently reviewed in a blinded comparison of AI and faculty feedback.

**Results:**

Twenty students submitted complete responses, generating feedback for a total of 80 questions. Mean AI feedback generation time was 34 s per quiz, compared with 508 s for faculty marking, representing a 15-fold reduction. Twelve of 20 (60%) feedback files required no modification before release, while eight required amendments, including four major corrections, even with faculty modification there was substantial efficiency gains from AI generated feedback. Eleven students (55%) completed the survey, reporting favourable perceptions of clarity, actionability, confidence and overall usefulness (median 4 out of 5) for the moderated feedback. In an exploratory blinded review of 5 double-marked submissions, no statistically significant differences were detected between AI-generated and faculty-generated feedback across rated domains (*p* > 0.23 for all domains).

**Conclusions:**

A custom GPT-based LLM delivered rapid, structured pharmacology feedback with substantial efficiency gains and positive student perceptions. However, clinically important errors occurred, necessitating consistent faculty oversight. Generative models may augment formative assessment in medical education, but rigorous calibration, moderation and ethical safeguards remain essential for safe implementation.

**Supplementary Information:**

The online version contains supplementary material available at 10.1186/s12909-026-09300-w.

## Practice points

- Raw AI feedback generation was rapid (≈34 seconds per four-question quiz vs ≈8.5 minutes for fully manual faculty marking), although safe deployment still required faculty review.
- Most outputs were usable with no or only minor edits.
- Students rated moderated AI feedback highly (median 4/5).
- In a small exploratory blinded comparison, moderated AI feedback received similar domain ratings to faculty feedback, although the sample was too small to support equivalence claims.
- Consider use of rubrics, references, and ongoing human oversight for safe rollout.

## Introduction

Timely, individualised feedback is important to promote reflective learning and developing core knowledge. Formative assessment that provides constructive feedback helps support students to realise their learning goals [1]. A recent study from over 6,000 medical students demonstrated that teacher feedback is vital in fostering their independent learning skills [2]. However, writing and providing feedback from short answer question (SAQ) style tests can be labour intensive on faculty workload due to the nature of the free text responses in comparison to single-best answer (SBA) exams. Unlike SBA questions, SAQs have the advantage of allowing assessment of mechanistic reasoning and applied knowledge. Large language models (LLMs) trained on reliable information can generate coherent prose and have demonstrated value in creating learning outcomes and assessment items [3].

Recent studies have shown that LLMs can grade SAQs with reasonable agreement to faculty markers although the quality and student-perceived utility of the feedback has not been rigorously nor prospectively assessed [4, 5]. Emerging work focused specifically on LLM-generated feedback suggests that these systems can produce structured, relevant and potentially useful feedback for learners, including in medical education settings [6, 7]. Related work has also demonstrated the potential for LLMs to apply analytic rubrics to other constructed-response assessment formats, such as in objective structured clinical examinations (OSCEs), supporting the broader relevance of rubric-guided LLM scoring in health professions education [8]. However, reported performance remains variable, with concerns relating to factual accuracy, hallucinations, pedagogical alignment, and whether feedback is sufficiently specific and actionable without expert review [9, 10]. A recent review has also highlighted that much of the literature remains early-stage and heterogeneous, with limited prospective evaluation [11]. These considerations support the need for studies that examine not only speed and acceptability, but also correctness, educational usefulness and the role of human moderation in practice.

The primary aim of this pilot study was to explore and begin to address these gaps by developing a custom GPT-based feedback tool for pharmacology SAQ feedback, unlike prior studies using generic AI tools [12]. The objectives were to assess the feasibility and accuracy relative to faculty marking, the correctness of the feedback and to explore students’ perception of faculty-reviewed AI-generated feedback. We hypothesised that the model would deliver structured and timely feedback and that students would find it useful but that faculty moderation would remain necessary to ensure accuracy.

## Methods

### Ethical approval, study design & setting

The study protocol was approved by our institutional research governance and ethics committee (committee reference number: ER/BSMSA24E/1). Participation was voluntary and students provided written informed consent electronically prior to enrolling in the study.

**Fig. 1:**
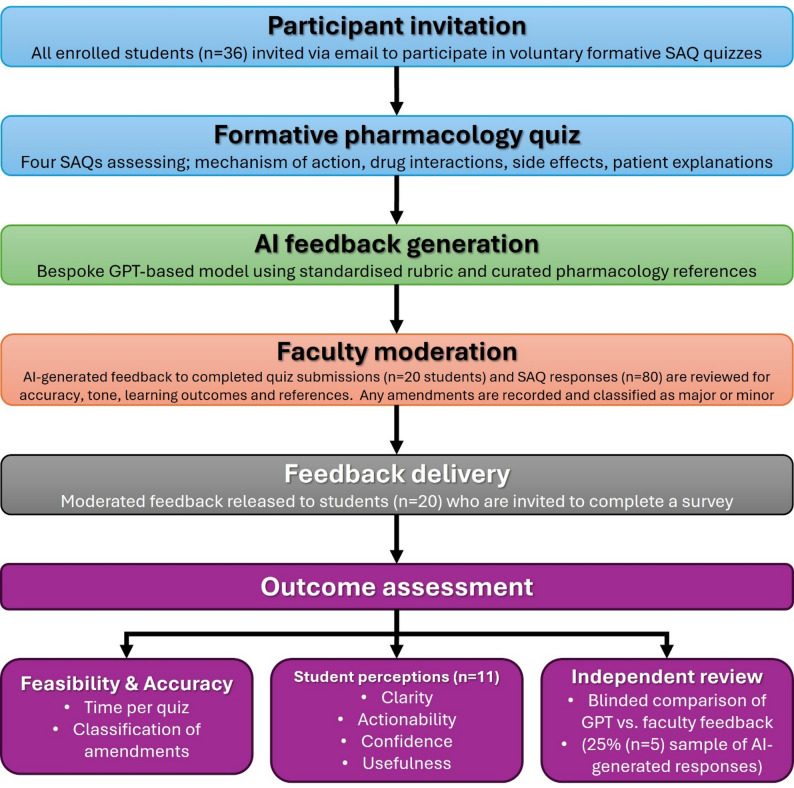
Study workflow for the delivery and evaluation of AI-generated formative pharmacology feedback. Thirty-six enrolled students were invited by email to participate in voluntary formative SAQ quizzes. Twenty students completed four-question quizzes, yielding 80 individual SAQ responses. These were processed using a bespoke GPT-based model with a standard rubric and curated pharmacology references. Twenty AI-generated feedback files, one per student submission, underwent faculty moderation before release. Outcomes were assessed across feasibility and accuracy, student perceptions and independent blinded comparison of GPT and faculty generated feedback of a 25% sample

We conducted this pilot study in the Physician Associate (PA) studies Master of Science (MSc) degree at our institution from March to October 2025. In the UK, PA programmes are postgraduate professional training courses preparing students for clinical practice in a supervised medical role. Participants in this study were students undertaking training within this MSc programme, with pharmacology taught as part of our core curriculum. The intervention was integrated into a series of formative pharmacology assessments consisting of four SAQs covering drug mechanism of action, drug interactions, side effects and patient explanations (example questions are provided in Supplementary Table 1). Completion of the quizzes was voluntary and did not contribute to summative results. After submission and in accordance with the study protocol, all AI-generated feedback was reviewed by faculty members before release to students, and learners received only the moderated final version. Anonymised datasets used and/or analysed during the study are available from the corresponding author upon reasonable request. The overall study workflow is summarised in Fig. 1.

### Participants

All PA students enrolled on the course during the period of the study (*n* = 36) were invited to participate via an email invitation. This included hyperlinks to SAQ-style tests on individual pharmacology topics that had been covered during the pharmacology module on our course. All students who completed the quizzes were sent faculty reviewed AI-generated feedback and invited to provide their feedback on this moderated feedback as well. The participant information sheet and consent form explicitly stated that AI-generated feedback would be reviewed by a member of faculty prior to release. The faculty moderator was an experienced pharmacology educator involved in the delivery of the PA curriculum and familiar with the assessment rubric.

### Intervention model

We developed a bespoke custom generative pre-trained transformer (GPT)-based model hosted on the ChatGPT platform configured specifically for this project. The custom GPT was built using OpenAI GPT-4-family large language models (primarily GPT-4o and GPT-4.1 variants), with detailed system-level instructions designed to support consistent and structured feedback generation, the system prompt is provided in Supplementary Materials.

The GPT was programmed to generate feedback using a standardised rubric which assessed four pharmacology-relevant domains: mechanism of actions, drug interactions, side effects and patient explanation. This rubric was embedded within the model’s operational instructions and used to guide scoring and feedback (Supplementary Table 2). In addition, the custom GPT was provided with curated pharmacology reference materials, including SmPC-derived content for the top 100 drugs covered in our pharmacology teaching, based on the published UK starter formulary for trainee prescribers and sourced from the UK electronic Medicines Compendium (emc), a publicly accessible repository of regulatory-approved product information [13, 14].

No personal identifiers were included in any uploaded data and when presented with a document containing student responses, the GPT was instructed to complete feedback without additional prompting once the document was uploaded with an initial, standardised prompt. To ensure uniformity, the GPT was constrained to provide similarly structured components of feedback, explicitly correcting misconceptions and providing references for any major corrections. In accordance with the study protocol, all GPT generated feedback was reviewed by faculty members before release to students. The feedback was assessed for factual accuracy, alignment with learning outcomes, appropriateness of tone and validity of references and any amendments were recorded as a measure of the GPT accuracy.

### Outcome measures

Primary outcomes were feasibility and accuracy. Feasibility was assessed by prospectively timing three workflow components: (1) AI feedback generation per four-question SAQ quiz, measured in real time using a timer from entry of the student response with the standardised prompt to completion of the GPT-generated feedback output; (2) fully manual faculty marking and feedback generation per four-question quiz, measured in real time using a timer from the start of review of the student submission to completion of the written feedback; and (3) faculty review and amendment time for AI-generated feedback prior to release. Faculty timing data were recorded by a single faculty marker. Accuracy was defined as the number and classification of amendments following faculty moderation. Amendments were classified as major if they involved clinically unsafe, materially misleading, or educationally significant errors requiring substantive correction before feedback could be released to students. This included errors such as incorrect drug-safety advice, inaccurate counselling likely to reinforce unsafe practice, or major factual inaccuracies affecting the core learning objective. Amendments were classified as minor if they improved precision, completeness, scoring consistency, or mechanistic detail without altering the overall safety of the feedback or introducing materially misleading guidance. Amendment severity was recorded by the faculty moderator during the moderation process at the time each feedback file was reviewed. Student perceptions were captured via a 5-point Likert scale survey measuring clarity, actionability, impact on confidence and overall usefulness of the faculty-reviewed AI-generated feedback, plus optional free-text comments. The full questionnaire is provided in Supplementary Table 3.

Finally, to assess accuracy, 25% of the student responses were double marked by faculty. Then a researcher not involved in pharmacology teaching, marking or model development, and blinded to the source of the feedback, and independent from the pharmacology module reviewed the paired faculty and AI-generated feedback to assess for accuracy, depth, actionability and clarity via a 5-point Likert scale. The full blinded evaluation questions are provided in Supplementary Table 4.

### Statistical analysis

Time to marking was summarised using means. For each Likert-scale item we calculated the median with interquartile range (IQR) and counts for each rating (1–5). Free text comments were reported thematically to identify recurrent topics. For the exploratory blinded comparison, paired AI-generated and faculty-generated feedback ratings across four domains were compared using Wilcoxon matched-pairs signed-rank tests, with Holm-Šídák correction applied for multiple comparisons. Free-text comments were reported thematically to identify recurrent topics. Only aggregated, anonymised results are reported. Statistical analyses were performed using Prism (version 10.6.1, GraphPad Software, Boston, USA).

## Results

### Participants and dataset

Twenty students submitted complete SAQ responses with four questions each. Each student completed a set of four curriculum equivalent SAQs drawn from different individual pharmacology topics (e.g. cardiovascular pharmacology), resulting in 80 total AI-generated feedback outputs. Eleven students (55%) completed the optional post-feedback survey.

### Accuracy and error analysis

Of the 20 AI‑generated feedback files, 12 were released to students without modification. Eight files required amendments following faculty moderation. Four of the eight amended files (50%) were classified as major, typically involving omission of critical adverse‑drug reactions or contraindications. The remaining four amendments were minor, addressing overestimation of student performance or insufficient emphasis on mechanisms of action (Supplementary Table 5).

### Feasibility (Time to Mark)

The mean AI feedback generation time for a four-question SAQ quiz was 34 s (SD 5 s). By contrast, fully manual faculty marking and feedback generation required a mean of 508 s per quiz (approximately 8.5 min, SD 110 s). On this basis, raw AI generation was approximately 15 times faster than fully manual faculty marking. Across the full cohort of 20 submissions, AI generation required approximately 11 min in total, whereas fully manual faculty marking would require approximately 2.8 h. Faculty review and amendment of AI-generated feedback required 3,360 s in total (56 min; Supplementary Table 6). Therefore, the combined AI-assisted moderated workflow for the cohort required approximately 67 min in total.

### Student perceptions

**Fig. 2:**
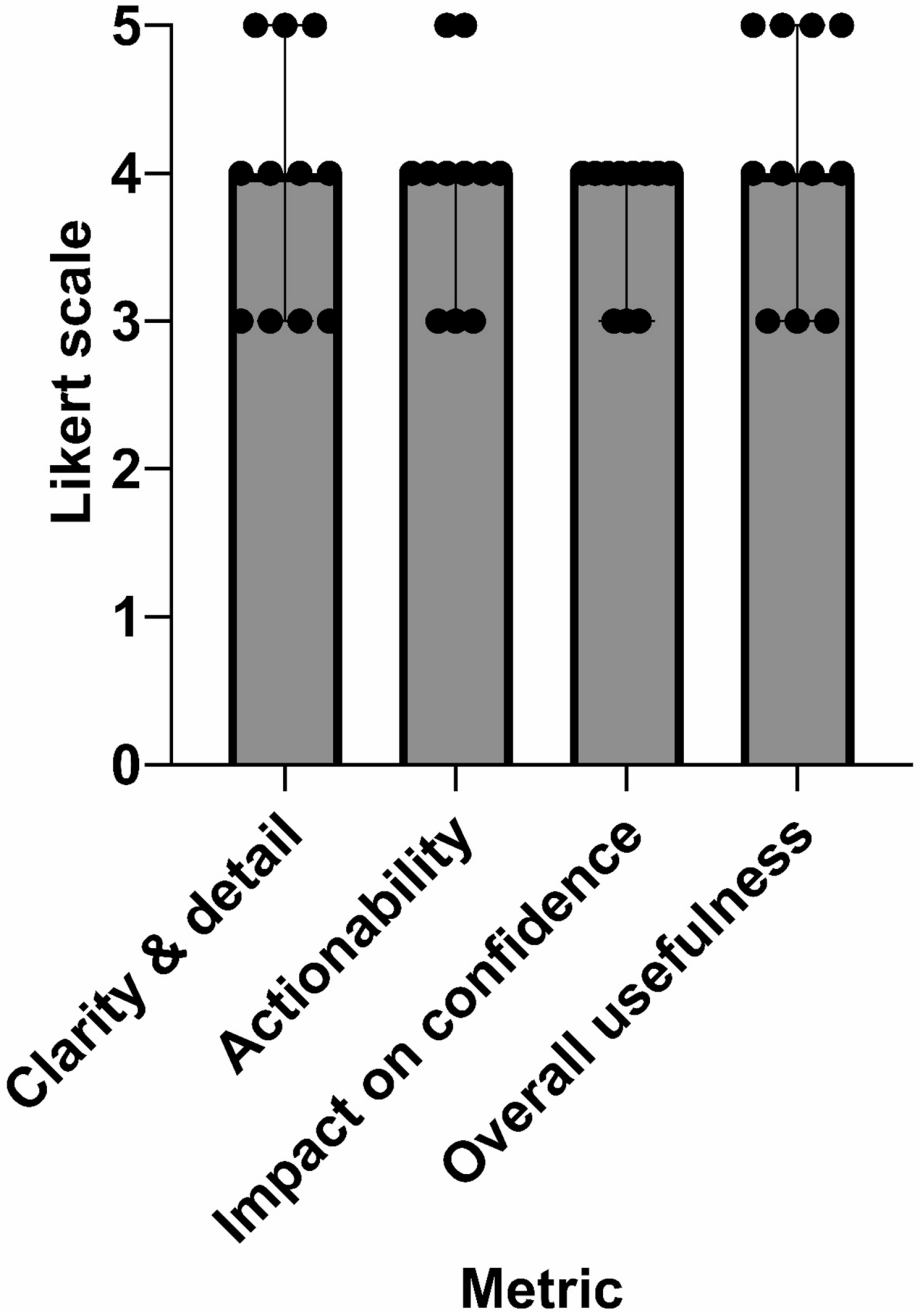
Distribution of student ratings across survey domains. The frequency of ratings (1–5, higher numbers indicating more favourable) for clarity, actionability, impact on confidence and overall usefulness, data presented as median ± IQR

Survey responses indicated favourable perceptions of the faculty-reviewed AI-generated feedback that was delivered to students (Fig. 2). Clarity received a median score of 4. Actionability, impact on confidence and overall usefulness yielded similar medians of 4, with most students rating the feedback 4 or 5. Distributions were skewed toward positive responses; no student rated any item below 3.

Free-text comments (*n* = 5) underscored the perceived fairness and transparency of the faculty-reviewed AI-generated feedback. Students appreciated explicit justification of marks, graphic icons, highlighting rubric alignment and clear explanations of how to improve their answers. Suggestions for improvement included providing model sentences as examples, bolding corrections to aid revision and listing common exam pitfalls.

### Faculty comparison

**Fig. 3:**
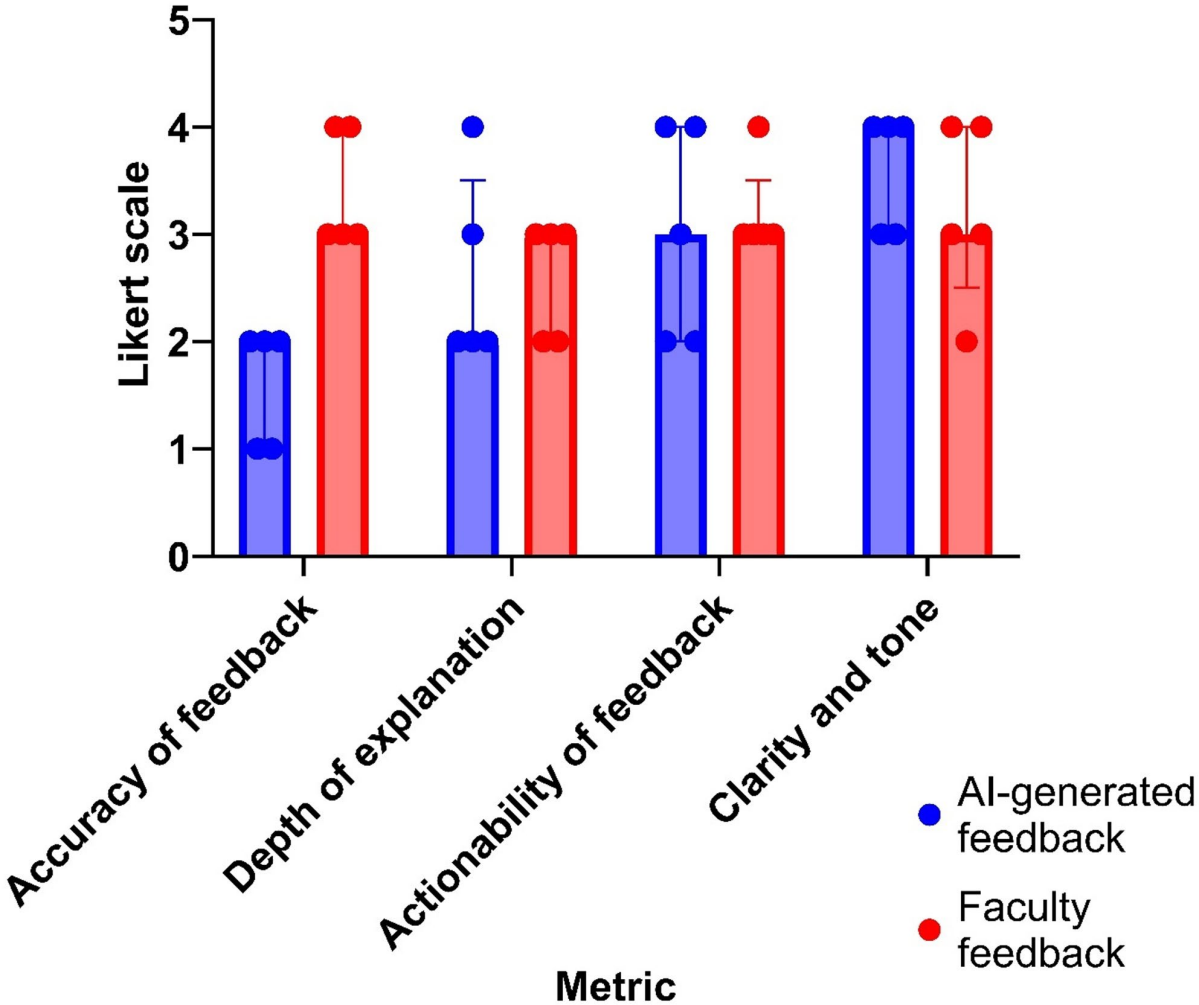
Distribution of blinded faculty analysis of student feedback across survey domains. The frequency ratings (1–4, higher numbers indicating more favourable) for accuracy of feedback, depth of explanation, actionability of feedback and clarity and tone with data presented as median ± IQR. No statistically significant differences were detected in this exploratory comparison of 5 double-marked submissions (Wilcoxon *p* > 0.23 for all domains)

The AI-marked results for 5 students (25%) were then also marked by a member of faculty and hence were double marked. An independent member of the research team then analysed these two pieces of feedback and assessed them for accuracy, depth, actionability and clarity. In this exploratory comparison of 5 double-marked submissions, no statistically significant differences were detected between AI-generated and faculty-generated feedback across rated domains (Wilcoxon *p* > 0.23 for all, Fig. 3). However, the comparison was based on a very small sample and should be interpreted cautiously. The blinded reviewer correctly identified all five AI-generated feedback items and four of the five faculty-generated feedback items, suggesting that perceptible differences between AI and faculty feedback remained.

## Discussion

This pilot study is the first to demonstrate that a custom GPT model can deliver structured, rubric‑aligned feedback on pharmacology SAQs with substantial efficiency gains over traditional marking. Among survey respondents, students rated the clarity, actionability and usefulness of the faculty-reviewed AI-generated feedback highly, and free‑text comments highlighted transparency and fairness. Despite positive perceptions, nearly half of the AI outputs required faculty moderation before being released to students, with major errors involving omission of critical safety information. The AI tended to award slightly generous scores and occasionally under‑emphasised mechanistic reasoning. These findings suggest that generative models can augment faculty assessment efforts, but this must be carefully calibrated and supervised to ensure accuracy.

Our results align with prior studies demonstrating the potential of LLMs to support medical education. Recent studies have shown that generative AI tools can produce learning outcomes and assessment items, and that LLMs can grade short-answer questions comparably to expert markers, though oversight is needed and performance varies by discipline [3, 5]. Related work in OSCE assessment has also suggested that AI may serve as a supplementary evaluation tool, with stronger performance in more visually observable tasks [15]. Another recent study found LLM feedback from multiple-choice questions to be overall rated as useful by participants despite noting inaccuracies being generated occasionally [16]. Whereas, generic AI use has shown benefits, we also highlighted the need for faculty moderation of bespoke AI pharmacology feedback [12].

Our findings extend this literature by demonstrating that a custom GPT-based tool can provide rapid SAQ feedback within a pharmacology curriculum, combining speed advantages with student acceptability. They also reinforce cautions from recent Association for Medical Education in Europe (AMEE) Guides that AI research must transparently report methods and include human oversight to mitigate hallucinations and ethical risks [17, 18].

This study offers several strengths. First, the custom GPT was configured using authoritative pharmacology sources and a faculty-designed rubric, promoting alignment between feedback and curricular learning objectives. Second, prompts enforced a structured output with explicit scoring and explanatory bullet points, which likely contributed to positive student perceptions. Third, safeguards such as de‑identification, fixed feedback length and mandatory citations were implemented, addressing ethical concerns.

In terms of limitations, the pilot nature and small sample size limit generalisability. No formal a priori sample size calculation was performed, as the study was designed as a pilot evaluation intended to assess feasibility and generate preliminary data. Only 20 students participated, and survey responses were obtained from 11, introducing potential response bias. As completion of both the formative quizzes and post-feedback survey was voluntary, students who found the feedback more useful or engaging may have been more likely to respond. Error classification relied on researcher judgement, and the threshold for major versus minor amendments may not be generalisable.

The major-error rate in this pilot also requires careful interpretation. Four of 20 feedback files required major correction before release and the major corrections in this pilot appeared to cluster around error types that may be relevant for safe implementation. First, some outputs appeared to insufficiently challenge incorrect assumptions within the student response and instead generated feedback within that flawed frame, risking reinforcement of misconceptions. Second, several major corrections involved omission of safety-critical contextual advice, particularly around medicine counselling and prescribing exceptions. Third, one error involved inaccurate identification of the components of a combination inhaler, illustrating the potential for mistakes in drug-product knowledge even when the broader therapeutic class is correctly recognised. In contrast, minor corrections more commonly involved overestimation of student performance or insufficient mechanistic depth. The efficiency gains reported here therefore apply to a moderated workflow rather than autonomous AI deployment. Although faculty review remained substantially faster than fully manual marking, the review process still required meaningful faculty time.

The blinded comparison was conducted by a single independent reviewer, which may have introduced subjectivity and these findings should therefore be interpreted cautiously. The blinded comparison should be interpreted as exploratory only, as it included five double-marked submissions and was underpowered to detect meaningful differences; accordingly, the absence of statistically significant differences should not be taken as evidence of equivalence. Finally, as with all AI applications, the risk of hallucination persists, and regular retraining and monitoring are required such as the safeguards proposed in our pilot study.

The substantial reduction in marking time illustrates the potential of generative models to alleviate faculty workload and provide prompt feedback. In time-pressured higher education environments, such efficiency gains may allow redistribution of faculty effort towards higher-value educational activities such as mentoring, personalised academic support or supporting communication skills in healthcare related studies. Furthermore, by delivering prompt, structured feedback, generative models may support learners’ goal‑setting and reflection. However, because self-regulated learning is influenced by the quality of feedback, the necessity of expert moderation in our study aligns with evidence that effective feedback requires contextualisation and interpersonal engagement. Additionally, by structuring feedback around a rubric, AI can support consistency and transparency in assessment, which students appear to value.

Editorials have questioned whether medical educators are ready to embrace AI, citing risks of bias and misalignment with curricular goals [19]. Institutions considering implementation should invest in model calibration, training of faculty moderators and ongoing evaluation. With these caveats, adoption of LLMs in medical education may allow faculty to redirect time towards higher‑level tasks, such as mentoring and curriculum development. Even where AI tools are not adopted, structured rubric aligned feedback with explicit justification of marks and clear guidance for improvement enhances feedback transparency. The structured format developed for this study could inform faculty marking proformas or assessor training to promote consistency, particularly in SAQ feedback.

Larger studies across multiple institutions and disciplines are needed to evaluate generalisability and to determine whether AI‑generated feedback translates into improved learning outcomes. Comparative trials should examine different LLM models and explore reinforcement learning from human feedback to reduce error rates and bias. Longitudinal designs could assess how repeated exposure to AI feedback affects knowledge retention, clinical reasoning along with summative assessment results. Finally, ethical frameworks should be refined to balance efficiency with safety, accountability and fairness, particularly as generative models become increasingly sophisticated.

In conclusion, a custom GPT‑based model delivered rapid, structured feedback on pharmacology SAQs, significantly reducing marking time and receiving favourable student evaluations. However, expert moderation remained critical, particularly to identify uncorrected misconceptions, missing safety advice, and clinically important pharmacology errors. Generative models may hold promise as tools to augment educational feedback, but rigorous calibration, ethical safeguards and human oversight are essential for responsible integration into medical curricula.

## Supplementary Information

Supplementary Material 1.

Supplementary Material 2. Supplementary Table 1: Example short answer questions (SAQs) for various pharmacology specialty topics. Supplementary Table 2: Marking rubric for short answer questions (SAQs). Supplementary Table 3: Blinded evaluation tool used for independent comparison of AI-generated and faculty-generated feedback. Supplementary Table 4: Student feedback questionnaire used to evaluate perceptions of the feedback provided. Supplementary Table 5: Examples of major and minor discrepancies between AI-generated marking and faculty review of student SAQ responses. The table summarises overestimation, factual inaccuracies and unsafe counselling advice, with recommended score adjustments and severity grading. Supplementary Table 6: Faculty time required to review and, where necessary, amend AI-generated feedback before release to students. Individual review times per student submission are shown in seconds for 20 submissions.

## Data Availability

Anonymised datasets used and/or analysed during the study are available from the corresponding author on reasonable request.

## Abbreviations

AI: Artificial intelligence
AMEE: Association for Medical Education in Europe
GPT: Generative pre-trained transformer
IQR: Interquartile range
LLM: Large language model
OSCE: Objective structured clinical examination
PA: Physician Associate
SAQ: Short answer question
SBA: Single best answer
SmPC: Summary of product characteristics
