# Supplementary material for: Artificial intelligence-assisted feedback in pharmacology education: a pilot evaluation of a custom generative model
Source: BMC Med Educ. 2026 May 30;26:1238. doi: 10.1186/s12909-026-09300-w (PMC13435439; doi:10.1186/s12909-026-09300-w)
Supplement: Supplementary file 1 [file 12909_2026_9300_MOESM1_ESM.docx]

**Supplementary Materials**

**Custom GPT prompt**

Standard Opening Phrase:

"Dear Student, thank you for your answers. Please find your feedback below."

Repetition of the Question to provide context.

Student’s Answer copied exactly, never truncate or abbreviate their answer.

Detailed Feedback for each answer:

✅ If correct, reinforce understanding.

❌ If incorrect, explain the mistake AND provide the correct information.

⚠️ Suggest improvements and clarify any missing details.

⭐ Use bullet points and appropriate emojis for clarity.

Ensure all students receive feedback of equal depth and length.

Score for Each Question, with clear justification based on rubric criteria.

Overall Score for the Student stated at the end.

Global Feedback, summarizing strengths and areas for improvement (without the student’s name).

References for Each Correction, citing sources such as the BNF, Rang & Dale, or SmPCs. Each major correction must have a supporting reference.

Provide all student feedback in a single response—no additional prompting is ever required, continue until all students have replies from the uploaded document.

🚨 Key Principles:

No student is skipped or given less detailed feedback.

All students receive constructive criticism and positive reinforcement.

Incorrect answers are always explained, not just marked wrong.

Scoring must be consistent and justified.

Every correction should include an appropriate reference
Below is first set of answers for feedback:

**Example LLM feedback:

Feedback not requiring amendment**

**❓ Question 2: A 60-year-old man on simvastatin is prescribed clarithromycin for a respiratory infection. Explain why this combination may be problematic and suggest an alternative approach**

**Your Answer:** 
*Clarithromycin inhibits CYP3A4, raising simvastatin levels and the risk of myopathy/rhabdomyolysis. Options: pause simvastatin during the antibiotic treatment, switch the statin to pravastatin/rosuvastatin, or choose a non-interacting antibiotic such as doxycycline.*

**Feedback:**

✅ **Correct interaction mechanism** – Clarithromycin inhibits **CYP3A4**, leading to **reduced metabolism of simvastatin** and a significant **increase in plasma levels**.

✅ **Adverse effects clearly identified** – You correctly note increased risk of **myopathy and rhabdomyolysis**, which is dose-related and potentially serious.

✅ **Alternative strategies all appropriate**:

- **Pausing simvastatin temporarily** is a short-term, safe approach.
- **Switching statin** (e.g., to **pravastatin or rosuvastatin**) is a sound long-term strategy.
- **Changing the antibiotic** (e.g., to **doxycycline**) avoids the interaction entirely.

⭐ A model response that demonstrates pharmacological accuracy and clinical judgment.

**Score:** **4/4** 
**Rubric Justification:** Comprehensive and clinically sound; addresses mechanism, risks, and alternatives effectively.

📘 **References:**

- BNF: Simvastatin-clarithromycin interactions
- SmPC: Simvastatin
- NICE CKS: Lipid-modification – CVD prevention

**Feedback requiring amendment

❓ Question 4: How would you explain the use of a combination inhaler (LABA + ICS) to a patient newly prescribed Fostair for asthma control?**

**Your Answer:** 
*It’s a mix of beclometasone with salmeterol that you use with asthma. Take a couple of puffs when needed and it works well with your relieve (blue) inhaler.*

**Feedback:**

❌ **Poor explanation of role** – The purpose of ICS (anti-inflammatory) and LABA (long-acting bronchodilator) should be clearly explained.

💡 Better explanation: “Fostair combines a steroid (beclometasone) to reduce airway inflammation and a long-acting bronchodilator (formoterol) to keep airways open. It should be taken every day as prescribed, and you should rinse your mouth after using it. Keep your blue inhaler for sudden symptoms.”

**Score:** **2/4** 
**Rubric Justification:** Poor patient counselling; medication details and use incorrect.

📘 **References:**

- BNF: Fostair
- NICE CKS: Asthma Management

**Amendments added:**
❌ **Wrong LABA** – Fostair contains **formoterol**, not **salmeterol**.

❌ **Incorrect usage advice** – Fostair is typically prescribed as a **maintenance inhaler** (taken **regularly**, e.g., **twice daily**) and not just “when needed”, unless it is part of a specific **MART** regimen (Maintenance and Reliever Therapy).
