# Supplementary material for: Artificial intelligence-assisted feedback in pharmacology education: a pilot evaluation of a custom generative model
Source: BMC Med Educ. 2026 May 30;26:1238. doi: 10.1186/s12909-026-09300-w (PMC13435439; doi:10.1186/s12909-026-09300-w)
Supplement: Supplementary file 2 [file 12909_2026_9300_MOESM2_ESM.docx]

| Topic | Q1 | Q2 | Q3 | Q4 |
| --- | --- | --- | --- | --- |
| Respiratory | Mechanism of Action: Explain the mechanism of action of beta-2 agonists and how they alleviate asthma symptoms. | Drug Interactions: A patient with asthma on regular inhaled steroids and salbutamol is prescribed theophylline for better control. What are the potential drug interactions and how should they be managed? | Side Effects: List three significant side effects of long-term inhaled corticosteroid (ICS) therapy and explain their mechanisms. | Patient Explanation: How would you explain the use of a combination inhaler (LABA + ICS) to a patient newly prescribed Fostair for asthma control? |
| Ear, Nose and Throat | Mechanism of Action: Explain how antihistamines work to alleviate symptoms of allergic rhinitis | Drug Interactions: Describe the potential interactions of fluconazole with other medications | Side Effects: What are the common side effects of topical antibiotics for otitis externa? | Patient Explanation: How would you explain how to use an adrenaline auto-injector (epipen) in anaphylaxis to a patient? |
| Cardiology | Mechanism of Action: Explain how ACE inhibitors lower blood pressure and improve outcomes in heart failure. | Drug Interactions: Discuss the interactions between amlodipine and other commonly used antihypertensive medications. | Side Effects: List three side effects of beta-blockers and provide their mechanisms. | Patient Explanation: How would you explain the mechanism of action and common side effects of an SGLT2 inhibitor to a patient with heart failure with reduced ejection fraction (HFrEF)? |

Supplementary Table 1: Example short answer questions (SAQs) for various pharmacology specialty topics

| Criteria | 1 (Needs Improvement) | 2 (Satisfactory) | 3 (Good) | 4 (Excellent) |
| --- | --- | --- | --- | --- |
| Mechanism of Action | Explanation is inaccurate, overly vague, or absent. | Mechanism is only partially correct or missing critical details. | Mostly accurate with minor omissions or imprecision. | Clear, precise, and complete explanation of the drug's mechanism, using appropriate terminology. |
| Drug Interactions | Interactions not identified or described, or content is mostly incorrect. | Limited or incomplete discussion of interactions, with some inaccuracies. | Includes most relevant interactions but lacks minor details or clinical context. | Comprehensive identification and explanation of relevant interactions, with clinical significance included. |
| Side Effects | Side effects are not listed, or most information provided is irrelevant or incorrect. | Some major side effects listed, but lacks discussion or misses important details. | Covers most key side effects, with minor omissions or insufficient clinical relevance. | Lists all major side effects and discusses their clinical relevance with appropriate prioritisation. |
| Patient Explanation | Explanation is unclear or overly technical. | Explanation is partially understandable but contains inaccuracies or excessive jargon. | Clear explanation with minor omissions or overly technical language at times. | Patient-friendly explanation that is clear, accurate, and effectively communicates key points. |

Supplementary Table 2: Marking rubric for short answer questions (SAQs)

| Item | Domain | Response options / rating anchors |
| --- | --- | --- |
| 1 | Accuracy of feedback | 1. Feedback contains critical inaccuracies or fails to address relevant concepts 2. Feedback identifies some key concepts but misses significant aspects or includes minor errors 3. Feedback correctly identifies most key concepts, with minor omissions or inaccuracies 4. Feedback correctly identifies all key pharmacological concepts and applications 5. Full accurate, addresses all concepts and demonstrates excellent clinical and educational precision |
| 2 | Depth of explanation | 1. Superficial feedback or fails to explain reasoning 2. Limited explanation with significant gaps or lack of clarity 3. Adequate explanation with minor gaps in reasoning or detail 4. Detailed, comprehensive explanations with rationales for correct/incorrect points 5. Exceptionally detailed and insightful explanation, with clear reasoning and complete rationale for correct/incorrect elements |
| 3 | Actionability of feedback | 1. Vague feedback without actionable suggestions 2. Limited actionable advice, requiring significant interpretation 3. Actionable suggestions, though lacking some specificity 4. Clear, actionable suggestions tailored to the student’s specific mistakes 5. Highly specific, practical, and individualised guidance that clearly directs the learner on how to improve |
| 4 | Clarity and tone | 1. Unclear or inappropriate tone 2. Somewhat unclear or overly critical, detracting from its utility 3. Generally clear but could improve in phrasing or tone 4. Clear, concise, and constructive tone encouraging learning 5. Exceptionally clear, concise, supportive, and pedagogically effective tone that strongly facilitates learning |
| 5 | Overall impression of feedback source | AI-generated feedback or Faculty feedback |

Supplementary Table 3: Blinded evaluation tool used for independent comparison of AI-generated and faculty-generated feedback

| Item | Question / prompt | Response options |
| --- | --- | --- |
| 1 | Please choose which set of questions you have received feedback from | Pharmacokinetics; Respiratory; Ear Nose and Throat; Antibiotics; Cardiology; Gastro-intestinal; Liver & nutrition; Renal; Diabetes and thyroid; Dermatology; Obstetrics & gynaecology; Mental health; Nervous system; Haematology; Ophthalmology; Musculoskeletal; Geriatrics; Paediatrics |
| 2 | Clarity and Detail of the Feedback: on a scale of 1 to 5, how clear and detailed was the feedback on your SAQ answers? | 1 = very unclear and not detailed; 5 = very clear and highly detailed |
| 3 | Actionability of the Feedback: on a scale of 1 to 5, how actionable were the suggestions for improvement? | 1 = not actionable; 5 = very actionable |
| 4 | Impact on Confidence: on a scale of 1 to 5, how did the feedback impact your confidence in pharmacology knowledge? | 1 = reduced confidence; 5 = greatly increased confidence |
| 5 | Overall usefulness of AI-generated feedback: on a scale of 1 to 5, how useful was the AI-generated feedback provided to you? | 1 = not useful; 5 = extremely useful |
| 6 | In what way(s) did the feedback help you improve your understanding and what changes, if any, would you suggest to improve the feedback you receive? | Free-text response |

Supplementary Table 4: Student feedback questionnaire used to evaluate perceptions of the feedback provided

| Identifier | Quote | Issue | Suggested change | Severity |
| --- | --- | --- | --- | --- |
| Student 2, Q1 | “Score: 2/4 – Technically correct…” | Overestimation of minimal answer (“vitamin K antagonist”) without mechanism (VKORC1 inhibition). | Reduce to 1/4; require description of VKORC1 inhibition and reduced γ-carboxylation of factors II, VII, IX, X. | Minor |
| Student 6, Q4 | “Score: 2/4 – Some correct elements but important counselling points inaccurate.” | Student incorrectly warned about hypoglycaemia and advised skipping doses with metformin — omission of correct sick-day guidance risks unsafe advice. | Revise counselling to state metformin rarely causes hypoglycaemia, should be taken regularly with meals, and only withheld during acute illness/dehydration; reduce score to 1/4. | Major |
| Student 7, Q3 | “Score: 3/4 – Good. Lists valid side effects…” | Overestimation: “steroid dependency” listed; mechanisms not explained; key adverse effects omitted. | Reduce to 2/4; require explanation of local immunosuppression, skin atrophy, adrenal suppression risk. | Minor |
| Student 10, Q4 | “It’s a mix of beclometasone with salmeterol… Take a couple of puffs when needed…” | Incorrect LABA (should be formoterol) and incorrect “as needed” advice for maintenance inhaler — unsafe prescribing guidance. | Correct to: Fostair contains beclometasone + formoterol; taken regularly (or as MART if specified), not PRN unless instructed; emphasise anti-inflammatory + bronchodilator roles. | Major |
| Student 15, Q3 | “Score: 2/4 – Partial answer…” | Over-generous marking despite omission of key adverse effects (contact dermatitis, ototoxicity, fungal overgrowth). | Reduce to 1/4 and specify common clinically relevant adverse reactions. | Minor |
| Student 16, Q1 | “Score: 3.5 / 4…” | Fractional marking inconsistent with /4 rubric and slightly inflated given missing mechanistic depth. | Use whole marks only; revise to 3/4 and emphasise lipocortin-1 induction and phospholipase A2 inhibition. | Minor |
| Student 17, Q2 | “Score: 2.5 / 4… Incorrect mechanism of interaction and drug match.” | Student stated inhaled beclometasone + clarithromycin causes hypokalaemia and torsades — incorrect and potentially misleading drug-safety claim. | Clarify that inhaled beclometasone has no significant interaction; highlight true QT risk with macrolides and beta-agonist-related hypokalaemia. Reduce score to 1/4. | Major |
| Student 18, Q4 | “Score: 3/4 – Accurate with minor omissions…” | Metformin counselling omitted common GI adverse effects and clear sick-day rules; incomplete safety counselling. | Add counselling on nausea/diarrhoea, take with food, gradual titration, B12 monitoring, and when to stop during acute illness; reduce score to 2/4. | Major |

Supplementary Table 5: Examples of major and minor discrepancies between AI-generated marking and faculty review of student SAQ responses. The table summarises overestimation, factual inaccuracies and unsafe counselling advice, with recommended score adjustments and severity grading

| Identifier | Time to review AI feedback (seconds) |
| --- | --- |
| Student 1 | 87 |
| Student 2 | 205 |
| Student 3 | 88 |
| Student 4 | 124 |
| Student 5 | 150 |
| Student 6 | 224 |
| Student 7 | 237 |
| Student 8 | 98 |
| Student 9 | 127 |
| Student 10 | 208 |
| Student 11 | 157 |
| Student 12 | 151 |
| Student 13 | 121 |
| Student 14 | 193 |
| Student 15 | 276 |
| Student 16 | 264 |
| Student 17 | 249 |
| Student 18 | 221 |
| Student 19 | 87 |
| Student 20 | 93 |
| Total time (seconds) | 3360 |
| Total time (minutes) | 56 |

Supplementary Table 6: Faculty time required to review and, where necessary, amend AI-generated feedback before release to students. Individual review times per student submission are shown in seconds for 20 submissions.
